# Clinical Utility of an Internet-Delivered Version of the Unified Protocol for Transdiagnostic Treatment of Emotional Disorders in Adolescents (iUP-A): A Pilot Open Trial

**DOI:** 10.3390/ijerph17228306

**Published:** 2020-11-10

**Authors:** Bonifacio Sandín, Julia García-Escalera, Rosa M. Valiente, Victoria Espinosa, Paloma Chorot

**Affiliations:** Facultad de Psicología, Universidad Nacional de Educación a Distancia (UNED), 20040 Madrid, Spain; bsandin@psi.uned.es (B.S.); jgarciaescalera@psi.uned.es (J.G.-E.); vespinosa36@alumno.uned.es (V.E.); pchorot@psi.uned.es (P.C.)

**Keywords:** transdiagnostic, iUP-A, i-CBT, AMTE, anxiety, depression, adolescents, online therapy

## Abstract

The Unified Protocol for Transdiagnostic Treatment of Emotional Disorders in Adolescents (UP-A) has been shown to be effective for reducing symptoms of anxiety and depression in adolescents with emotional disorders. Internet-delivered psychological treatments have great potential to improve access to evidence-based psychological therapy since they are associated with reduced human and economic costs and less social stigma. Recently, our group developed an online version of the UP-A (the iUP-A) for the treatment of emotional disorders in adolescents. The aim of this pilot trial was to test the clinical utility of the iUP-A in a small sample (*n* = 12) of adolescents with elevated anxiety and/or depressive symptoms. Intention-to-treat and completer analyses revealed pre- to post-intervention self-reported decreases of anxiety and depressive symptoms, anxiety sensitivity, emotional avoidance, panic disorder symptoms, panic disorder severity, generalized anxiety disorder symptoms, pathological worry, and major depressive disorder symptoms. We found high feasibility and acceptability of the program with all participants and responsible parents reporting an improvement in the adolescents’ ability to cope with emotions. Results suggest that the iUP-A may provide a new approach to improve access to treatment for anxious and depressive adolescents in Spain; however, further research must be conducted before firm conclusions can be drawn.

## 1. Introduction

Anxiety and depression in youth represent a growing public health concern. Epidemiological studies indicate that as many as 32.4% and 10.6% of adolescents have lifetime prevalence rates of anxiety and depressive disorders, respectively [1]. Anxiety and depression have been associated with severe impairment and increased risk for future psychopathological problems [2]. Subclinical symptoms of anxiety and depression are also very prevalent (32% and 29.2%, respectively) and have been related to functional impairment and suicidality [3]. In addition to the high prevalence of these emotional disorders, anxiety and depression are overlapping conditions throughout the lifespan, with rates of comorbidity as high as 75% in clinical samples [4]. Children and adolescents with anxiety and mood disorders also share a number of underlying vulnerability and maintenance factors, such as temperament (e.g., negative affect), difficulty regulating affect across emotions (e.g., behavioral avoidance), and clinical dispositions to experiment negative reactions to emotions (e.g., anxiety sensitivity) [5,6].

Evidence-based cognitive-behavioral therapy (CBT) for anxiety and depressive disorders has traditionally been disorder-specific, i.e., focused on treating one disorder at a time without paying therapeutic attention to comorbid conditions. In contrast, the transdiagnostic approach tries to address this limitation by focusing on the shared features of anxiety and depressive disorders rather than on the symptoms specific to each of these disorders [7]. In line with this perspective, transdiagnostic CBT (T-CBT) for emotional disorders has emerged as a new therapeutic approach that targets psychopathological processes common to both anxiety and depression. Addressing emotional disorders’ risk and maintenance factors during adolescence may also help prevent the development of anxiety and depression later in life. The efficacy of T-CBT for emotional disorders has been found to be high and comparable to disorder-specific CBT treatments’ efficacy in recent meta-analyses [8,9,10].

The Unified Protocols for Transdiagnostic Treatment of Emotional Disorders in Children (UP-C) and Adolescents (UP-A) are recent manualized treatments developed by Ehrenreich-May et al. [11] to address a broad array of emotional disorders’ symptoms in children and adolescents. Both protocols take a transdiagnostic approach by focusing on a set of core change principles including prevention of emotional avoidance (effortful engagement in experiencing intense emotions), enhancement of cognitive flexibility (evaluation of potentially problematic cognitions), and change of maladaptive action tendencies (encouraging youth to alter problematic behavioral patterns related to their emotional symptoms). These protocols place particular emphasis on emotion regulation since a goal of these treatments is to help patients to learn how to experience uncomfortable emotions and how to respond to them in more adaptive and non-avoidant ways. The UP-C and UP-A are flexible administered treatment protocols designed to improve emotion reactivity and emotion regulation and to ameliorate anxiety and depressive symptoms by means of an array of evidence-based treatment techniques (e.g., psychoeducation, behavioral activation, cognitive restructuring, etc.) that are applied to a range of emotions including fear, anxiety, sadness, and anger.

The UP-A has a growing body of data supporting its efficacy in treating anxiety and depressive disorders and symptoms in adolescent samples [8,9,10,11,12,13,14,15]. In a multiple-baseline trial with three adolescents, all participants experienced a reduction in the severity of their principal anxiety or depression diagnosis at post-treatment [12]. The study of Trosper et al. [15], an open trial with 12 adolescents, found significant reductions in the principal disorders’ severity from pre- to post-treatment, as well as improvements in adolescents’ emotion regulation according to parent ratings. In a most recent study, Ehrenreich et al. [13] demonstrated the efficacy of the UP-A in a randomized, waitlist-controlled trial including 51 adolescents with a primary diagnosis of an anxiety or depressive disorder. According to the authors, the UP-A outperformed the waitlist condition since significant treatment effects were found in favor of the UP-A on all outcome measures. Additionally, at post-treatment participants in the UP-A condition showed lower diagnostic severity and greater improvements according to clinician-rated measures compared to those in the waitlist condition.

Recently, our group has reported data concerning the Spanish validation of the UP-A adapted as a universal school-based prevention program of anxiety and depression [16,17]. In an open-trial including 28 adolescents, significant self-reported decreases from pre- to post-intervention were found in anxiety outcome measures [17]; in addition, this study also provided support for the acceptability and feasibility of the Spanish version of the UP-A. In a more recent publication using the Spanish UP-A, results of a randomized waitlist-controlled trial including 151 school adolescents revealed that those participants with greater baseline emotional symptoms in the UP-A group trended toward significantly greater decreases in depression symptoms compared to the control group [16].

Internet-delivered psychological treatments represent an emerging model of service delivery with the potential to improve access to evidence-based treatments for anxiety and depressive disorders [18]. It has been suggested that internet-based treatments have several advantages compared with traditional face-to-face interventions, including improved access to evidence-based treatments and cost-effectiveness [19]. As these authors suggested, the fact that patients can return to the program at their convenience to access treatment information may facilitate learning and retention. Additionally, patients in internet interventions may receive therapist support faster than they would in traditional face-to-face treatments and may feel less stigma than when attending traditional psychological therapy. Several reviews and meta-analyses have shown comparable reductions in anxiety and depression symptoms from internet-based CBT (iCBT) and face-to-face CBT in adults [18,20,21], children, and adolescents [22].

A transdiagnostic iCBT treatment (T-iCBT) was recently developed by Titov’s group to treat anxiety and depression [18]. In a similar vein, Botella’s group developed a T-iCBT protocol based on Barlow’s UP [23] designed to treat emotional disorders in adults [24]. Several meta-analyses have shown that T-iCBT protocols could be effective in the reduction of anxious and depressive symptomatology [9,25]. Recently, our group has developed an internet-based version of the UP-A (i.e., the iUP-A) [26]. As far as we know, this is the first T-iCBT treatment that targets anxiety and depression in adolescents, as well as the first adaptation of the UP-A into a web-based or online format.

The aim of this pilot trial was to test the clinical utility, feasibility, and acceptability of the iUP-A in a small sample of adolescents living in Spain. We hypothesized to find a significant change from pre- to post-treatment in: (a) primary outcome measures (general levels of anxiety and depression), (b) transdiagnostic variables (positive and negative affect, anxiety sensitivity, and emotional avoidance), and (c) disorder-specific measures (symptoms of anxiety and depressive disorders, panic disorder severity, pathological worry, and symptoms of social anxiety). Specifically, we hypothesized that participants would evidence a significant reduction in all outcome measures at posttreatment compared to pretreatment levels, except for positive affect where we expected to find a significant increase. A second aim of the study was to examine the feasibility and acceptability of the iUP-A and of the web platform that implements it, called Learn to Manage your Emotions (Aprende a Manejar tus Emociones; AMTE).

## 2. Materials and Methods

### 2.1. Participants

Families were recruited through information posted by the Universidad Nacional de Educación a Distancia (UNED) on Twitter and on the UNED’s website as well as through school counselors’ referrals. The inclusion criteria for the adolescents were: (a) 12 to 18 years of age; (b) meeting diagnostic criteria for any anxiety and/or depressive disorder or having subthreshold anxiety and/or depression symptoms based on the Mini International Neuropsychiatric Interview (MINI) [27,28] at assessment; (c) having an e-mail address and daily access to the internet through a computer or electronic tablet; (d) stability of psychotropic medications (if any) for at least three months; (e) Spanish proficiency.

Exclusion criteria were: (a) a diagnosis of a severe psychopathology such as psychotic disorder, bipolar disorder, severe depressive disorder, intellectual disability, severe learning disability, autism spectrum disorder, or substance dependence; (b) being at moderate or severe risk for suicide (based on the MINI); (c) on-going psychological treatment; (d) no informed consent.

**Figure 1:**
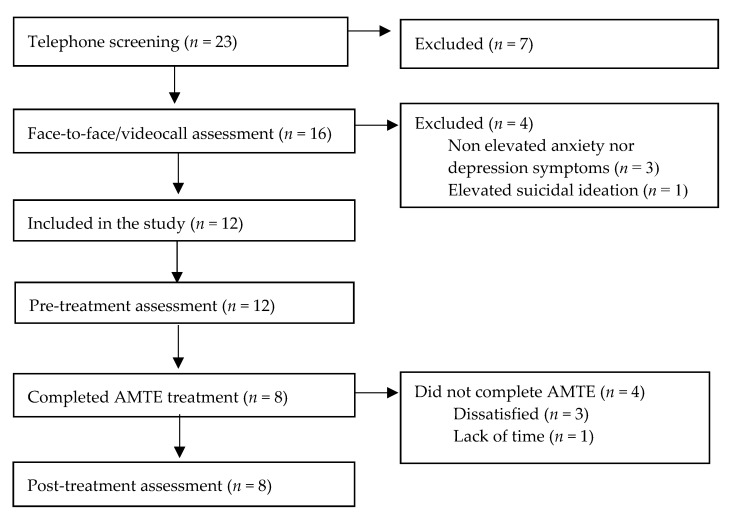
Participant flow through the study.

A total of 23 families were screened by telephone for eligibility. Participant flow through the study is presented in Figure 1. Post-treatment data were available for eight participants, all of whom completed the eight modules of the iUP-A. A total of four adolescents dropped out of the study, three reported being dissatisfied with the program whereas one reported a lack of time for the intervention. Of these four adolescents, two finished the first module, one finished the first four modules and one finished the first five modules.

A total of 12 adolescents met the inclusion criteria and were included in the study. There were eight girls (67%) and four boys (M age = 15.58 years old; SD = 1.73; range = 13 to 18). All adolescents were Spanish residents. Regarding the living situation, 10 adolescents (83.3%) were living with both of their parents whereas two were living with their mother and siblings due to parents’ divorce. In all cases (100%), the gender of the parent responsible for treatment was female.

None of the enrolled participants were taking psychotropic medication and only one had previously received psychological treatment (for a total of 10 sessions). Lastly, five adolescents (41.7%) were waiting for an appointment in the mental health department of the Spanish public healthcare system after being referred by their general practitioner or family doctor.

Regarding participants’ diagnoses, nine participants (75%) were diagnosed with some clinical disorder whereas three showed subthreshold anxiety and/or depressive symptoms (see Table 1).

### 2.2. Procedure and Design

This study employed an uncontrolled pre-post open trial design. It was granted ethical approval from the Research Ethics Committee of the UNED and was registered on Clinicaltrials.gov (Identifier NCT04182061). The TREND Statement guidelines for nonrandomized interventions [29] were followed when reporting the trial. No incentives were provided to the adolescents or parents for participating in this trial.

Recruitment was carried out in two steps: a telephone screening with the responsible parent and a face-to-face or video call assessment. Once the telephone screening had been made and the informed consent had been signed and returned, potential participants and their parents were invited to an assessment session that was conducted either face-to-face or through video call. In this session, a clinical psychologist conducted a full diagnostic interview with the adolescent and briefly talked with the parent(s) to gain insight regarding their concerns about the adolescents’ main problems and symptoms.

Following the assessment session, the adolescents meeting inclusion criteria were included in the study and both the responsible parent and the adolescent received an e-mail to register on the web platform Aprende a Manejar tus Emociones (AMTE) (Learn to Manage your Emotions). Then, one week before the intervention started, the adolescents were invited to fill in the self-report measures (see the Instruments section) on the AMTE platform. A week after the intervention ended, the adolescents were also invited to fill in the same self-report measures on the AMTE platform.

### 2.3. Intervention

The internet UP-A (iUP-A) was delivered by means of the AMTE platform. The iUP-A was adapted from the Unified Protocol for Transdiagnostic Treatment of Emotional Disorders in Adolescents (UP-A) [30].

As does the UP-A, the iUP-A uses evidence-based treatment techniques that cut across disorder-specific CBT treatment manuals for adolescent anxiety and depression (e.g., psychoeducation, cognitive restructuring, exposure, behavioral activation, relapse prevention, etc.). It also includes motivational enhancement as well as mindfulness-based techniques. The AMTE program includes the same eight core modules of the UP-A and participants are advised to complete one module per week (except for module seven, which usually takes one more week to complete). Table 2 provides an overview of the structure and specific topics address in the eight treatment modules.

The intervention can be used on (laptop) computers and tablets. Parents log in to a separate section of the program and have access to a summary of each module as well as to information regarding the progress of their adolescent in treatment. The therapists log in to a therapists’ section of the AMTE platform where they can monitor the progression of each adolescent in the treatment (last access to the platform, progression in the treatment modules, time spent on the platform, etc.) and see the adolescent’s answers to the home learning assignments.

**Figure 2:**
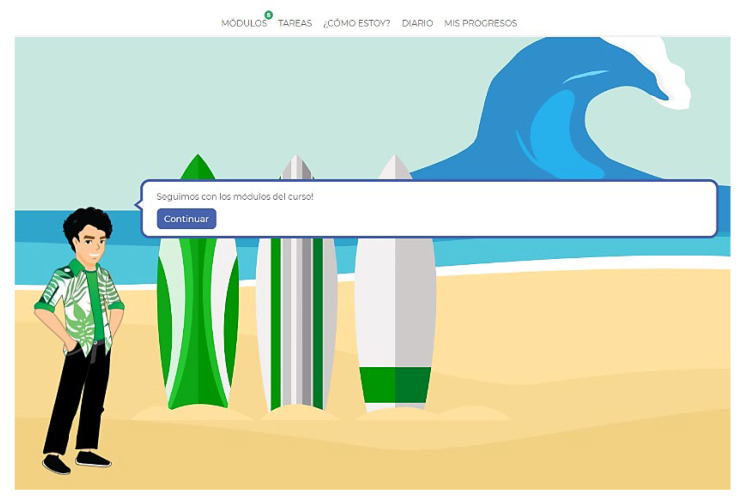
Screenshot of the AMTE homepage.

The treatment platform was designed with an age-appropriate appearance. The first time the adolescents log in, they personalize an avatar (virtual human) by changing its hair, skin color, clothing, and gender. The platform AMTE uses a narrator (called “Doctor”) that presents treatment contents through written messages whereas the avatar presents encouraging written messages to the participant. The AMTE platform uses an island overarching theme (see Figure 2).

Each module contains written texts presented by the Doctor or the avatar, explanatory videos, exercises, home learning assignments, and downloadable PDFs (with the exercises, the home learning assignments, and summaries of each module). The expected time to complete each module (contents, exercises, and home learning assignments) is approximately 50 min. The AMTE platform is interactive with responses to home learning assignments being directly entered into the program and saved so that the therapist can immediately review the adolescent’s progress. Participants may also initiate contact with the therapists by sending messages via the AMTE platform whenever they have questions or want to discuss any aspects of the treatment.

During the treatment period, both the adolescent and the treatment responsible parent had weekly telephone contact with their assigned clinical psychologist, the same one that did the face-to-face or videocall assessment. The psychologist called the responsible parent’s cell phone, spoke for about 10 min with the parent (to check on the parent’s main concerns regarding the adolescent and address any problems that may have arisen) and for another 10 min or so with the adolescent (to review the participant’s responses to the home learning assignments and support the adolescent in using the program).

### 2.4. Instruments

The clinical diagnoses were conducted by means of the MINI [27,28]. The questionnaires EAN [31], CDN [31], and the total score on the RCADS-30 [32] were used as primary outcome measures. To assess transdiagnostic constructs the questionnaires PANASN [33], CASI [34,35], and EASI-A [36,37] were used. Disorder-specific symptoms of anxiety and depressive disorders were assessed through the RCADS-30 subscales [32], and the questionnaires PSWQ-11 [38], SASC-R [39,40], and PDSS-SR [39,40,41,42]. Finally, the Feasibility and Acceptability Questionnaire (FAQ) was used to assess the experiences of the adolescents and parents with the program.

Mini International Neuropsychiatric Interview for Children and Adolescents (MINI-KID) [27,28]. The MINI-KID is a structured diagnostic interview for people aged 6 to 17 years. It is based on DSM-IV and ICD-10 criteria for psychiatric disorders. The reliability and validity of MINI-KID has been demonstrated [43].

Revised Child Anxiety and Depression Scale–30 (RCADS-30) [32]. The RCADS-30 is a 30-item self-report scale comprised of the following subscales derived from the Diagnostic and Statistical Manual of Mental Disorders (DSM-IV/5) criteria: (1) social phobia (Soc.P), (2) generalized anxiety disorder (GAD), (3) obsessive-compulsive disorder (OCD), (4) panic disorder (PD), (5) separation anxiety disorder (SAD), and (6) major depressive disorder (MDD). This scale has previously demonstrated good psychometric properties [7]. Each item is scored from zero (“never”) to three (“always”), with higher scores representing more severe symptoms.

Anxiety Scale for Children (Escala de Ansiedad para Niños, EAN) [31]. The EAN is a 10-item self-report scale that assesses anxiety symptoms in children and adolescents. It has shown good psychometric properties [16]. The participants are instructed to indicate how frequently they have experienced general anxiety symptoms (cognitive, physiological, etc.) on a four-point, Likert-type scale, ranging from zero (“never or almost never”) to three (“always or almost always”).

Depression Questionnaire for Children (Cuestionario de Depresión para Niños; CDN) [31]. The CDN scale is a 16-item, self-report questionnaire designed to assess symptoms of major depressive disorder and dysthymic disorder (according to DSM-IV/5 criteria) in children and adolescents. Participants rate each item on a four-point, Likert-type scale, ranging from zero (“never or almost never”) to three (“always or almost always”). The CDN has demonstrated good psychometric properties [16,32].

Positive and Negative Affect Schedule for Children and Adolescents (Escalas PANAS de Afecto Positivo y Negativo para Niños y Adolescentes; PANASN) [33]. The PANASN provides scores for two subscales of 10 items each measuring positive and negative affect. Participants are asked to rate items according to how they usually feel from one (“never or almost never”) to three (“a lot of the time”). This self-report scale has demonstrated adequate psychometric properties [32].

Childhood Anxiety Sensitivity Index (CASI) [34,35]. The CASI is an 18-item self-report questionnaire measuring anxiety sensitivity in children and adolescents, that is, distress reactions to symptoms of anxiety. Participants rate the frequency with which they experience each item using a three-point, Likert-type scale from one (“never”) to three (“a lot of the time”). The Spanish adaptation of the CASI used in the present study has demonstrated good psychometric properties in previous studies [44].

Emotional Avoidance Strategy Inventory for Adolescents (EASI-A) [36,37]. The EASI-A is a 17-item self-report questionnaire in which respondents are instructed to indicate the degree to which each statement is true using a five-point, Likert-type scale ranging from zero (“never or almost never”) to four (“always or almost always”).

PSWQ-11 questionnaire for children and adolescents (PSWQN-11) [38]. It is an age-downward version of the 11-item Penn State Worry Questionnaire [45]. This 11-item self-report questionnaire assesses pathological worry. Respondents are instructed to indicate the degree to which they agree with each statement using a five-point Likert-type scale from one (“Totally disagree”) to five (“totally agree”).

Social Anxiety Scale for Children-Revised (SASC-R) [39,40]. The SASC-R is an 18-item self-report questionnaire in which respondents are instructed to indicate the frequency with which they experience each symptom of social anxiety using a four-point Likert-type scale from one (“never”) to three (“a lot of the time”).

Panic Disorder Severity Scale—Self-Report (PDSS—SR) [41]. The abbreviated adapted version by Sandin (2010) [42] was used. The PDSS—SR is a seven-item self-report questionnaire that assesses the severity of the panic disorder in the last month. Using a five-point Likert-type scale from zero (“not at all”) to four (“very much”), participants rate the distress associated with panic attacks, anticipatory anxiety regarding new attacks, and interoceptive and situational avoidance. This scale has demonstrated adequate psychometric properties in adult samples [46].

Feasibility and Acceptability Questionnaire (FAQ) [47]. This questionnaire includes a version for the adolescents and a version for the parents. Both versions include three separate sections (subscales) each comprised of six items: (1) experience with the online platform, (2) satisfaction with the program, and (3) therapeutic alliance. All items are assessed on an 11-point scale from 0 (”totally disagree”) to 10 (”totally agree”). The parents’ version includes an additional section that assesses the experience of the treatment responsible parent with the parents’ section of the online platform. This section includes two items. The satisfaction with the program section of the FAQ includes the six questions from the satisfaction questionnaire of Rapee et al. (2006) [48].

### 2.5. Statistical Analysis

All statistical analyses were done with the SPSS v.24 software program (IBM Corp., Armonk, NY, USA). For the completer sample (*n* = 8), changes in the outcome scores between pre- and post-treatment were examined using the Wilcoxon signed-rank test, a nonparametric alternative to paired samples t-test. In addition to the completer analyses, calculations were repeated with the total sample (*n* = 12) after replacing missing values for the dropout cases with their last observation carried forward (LOCF). To gain further insight into the statistical significance of the improvements, effect sizes were calculated for all outcome measures using Cohen’s d (meanpre-meanpost/pooled SD) [49]. Before the statistical analysis of the data, we computed the statistical power (with an alpha error of 0.05, one-tailed) of the Wilcoxon signed-rank test (matched pairs) using the G*Power 3.1.9.7 [50]. The statistical power ranged from 75% to 100% (average = 96.7%) for the statistically significant outcome measures (described in Table 3 and Table 4); the power was 57% for the two marginally significant variables (SAD subscale of the RCADAS-30) and 87% PANASN-Positive Affect.

## 3. Results

### 3.1. Intervention Effects

A Wilcoxon matched-pairs signed-rank test including the completer sample, i.e., all the adolescents that finished the intervention (*n* = 8), showed a significant decrease in anxiety symptoms (EAN, *p* = 0.008) associated with a large effect size. Likewise, there were significant reductions in the RCADS-30 total score (*p* = 0.008), associated with a very large effect size. There were also significant reductions associated to several subscales of the RCADS-30: major depressive disorder (*p* = 0.031), panic disorder (*p* = 0.031), and generalized anxiety disorder (*p* = 0.016). Due to the small sample size, we also took into account marginally significant results (*p* < 0.10), which were found for the separation anxiety disorder subscale of the RCADS-30 (*p* = 0.063). Identical results were obtained including the intent-to-treat sample (*n* = 12); see Table 3.

Wilcoxon tests including the adolescents that finished the intervention (*n* = 8) showed significant reductions on the transdiagnostic outcome measures of anxiety sensitivity (CASI; *p* = 0.016) and emotional avoidance (EASI-A; *p* = 0.039), both associated with large effect sizes. Also, there were significant reductions in panic disorder severity (PDDSS-SR; *p* = 0.004) and pathological worry (PSWQ-N; *p* = 0.008), both associated with large within-group effect sizes. We also found that an increase in PANASN-PA from pre- to post-treatment was marginally significant (*p* = 0.094). Identical results were obtained including the intent-to-treat sample (*n* = 12); see Table 4.

### 3.2. Feasibility and Acceptability

#### 3.2.1. Adolescent Report

The FAQ was completed by eight adolescents (66.66% of those who participated in the program) on a scale from 0 to 10 (see Table 5). In relation to the questions assessing experience with the program, mean ratings ranged between 7.25 and 9.38. Regarding the satisfaction with the program, the mean ratings of the first four questions ranged between 8.50 and 9.38. In addition, all adolescents reported that their ability to cope with emotions had improved significantly (mean improvement from 4.13 to 8.25). Finally, ratings on the therapeutic alliance were high with mean ratings ranging from 8.88 to 9.88.

#### 3.2.2. Parents Report

The FAQ (parent version) was also completed by eight mothers (the mothers of the eight adolescents that finished the intervention) on a scale from 0 to 10 (see Table 6). In relation to the questions assessing the experience of the adolescent with the program according to the perception of the parent, mean ratings ranged between 8.38 and 9.25. Likewise, seven mothers (out of eight) reported having logged in to the family section of the platform at least once with the mean satisfaction with the platform being 7.29. Regarding the parent perception of satisfaction of the adolescents with the program, the mean ratings of the first four questions ranged between 8 and 9.75. Also, all mothers reported that the ability of their child to cope with emotions had significantly improved (mean improvement from 4.63 to 8.00). Finally, ratings on the therapeutic alliance were very high with mean ratings ranging from 9.38 to 9.88.

## 4. Discussion

The purpose of the current study was to examine the clinical utility of the UP-A adapted to an internet-delivered program (i.e., the iUP-A). We explored whether the iUP-A would significantly decrease, from pretreatment to posttreatment, the levels of (a) general measures of anxiety and depression, (b) transdiagnostic constructs (positive and negative affect, anxiety sensitivity, and emotional avoidance), and (c) disorder-specific measures (anxiety and depressive disorder symptoms, panic disorder severity, pathological worry, and social anxiety). We also examined the acceptability and feasibility of the iUP-A. As far as we know, this is the first study that examines the clinical utility of an online T-CBT program for the treatment of anxiety and depression in adolescents; thus, this is also the first evaluation of the UP-A implemented as a web-based intervention.

Consistent with our hypotheses, the participants of this study reported improvements in general symptoms of anxiety and depression, transdiagnostic measures, and disorder-specific symptomatology. According to the effect sizes’ estimates, the magnitude of the changes were medium (for positive affect and symptoms of MDD, social phobia, and separation anxiety disorder), large (for anxiety sensitivity, emotional avoidance and symptoms of general anxiety, panic disorder, GAD, and OCD), and very large (for panic disorder severity, pathological worry, and general symptomatology of anxiety and depression measured by RCADS-total score). Overall, we found significant or marginally significant improvements in all variables except for general symptoms of depression (questionnaire CDN), negative affect, social anxiety, and symptoms of OCD and social phobia. One surprising finding was the lack of significant effects on positive and negative affect. Although, according to the descriptive statistics, these temperamental variables changed from pretreatment to posttreatment as expected (the positive affect increased whereas the negative affect decreased), such changes did not reach the level of statistical significance.

The findings of this open trial are particularly noteworthy because the iUP-A appears to be effective at managing the three kinds of main variables examined (general symptoms of anxiety and depression, transdiagnostic measures, and specific symptoms of several anxiety and depressive disorders). Most of these positive effects were associated with large and very large effect sizes, which is consistent with results of the UP-A reported by Ehrenreich-May’s group [12,13,15], as well as with findings of our group related to the Spanish version of the UP-A [16,17]. These data are also consistent with the results reported by authors who administered Super Skills for Life, a T-CBT protocol that has been applied as an indicated prevention program for adolescents [51] and children [52] at risk of developing anxiety disorders.

The fact that the iUP-A was able to modify two major transdiagnostic variables (i.e., anxiety sensitivity and emotional avoidance) is an important strength of the present study. The core treatment modules of the UP-A, like the UP’s core modules [23], were designed to explicitly target aversive reactions to emotions (e.g., anxiety sensitivity) and the subsequent emotional avoidance. These are core transdiagnostic underlying processes related to the vulnerability and maintenance of emotional disorders and, therefore, processes shared by individuals with anxiety and depression. Surprisingly, there is a lack of evidence in the literature concerning the efficacy and clinical utility of the UP and UP-A reducing the levels of these main transdiagnostic mechanisms [10]. To our knowledge, this is the first study that provides empirical evidence of the UP-A’s ability to modify high levels of anxiety sensitivity and emotional avoidance in youth. The study of Calear et al. [53] used the same scale we used to measure anxiety sensitivity (i.e., the CASI) but did not obtain significant results. Concerning affectivity, although some studies have shown the ability of the UP to produce changes in negative affect (or neuroticism) and positive affect (or extraversion) [10,54] we did not find such an effect.

This is the first trial that examines the feasibility and acceptability of an internet-based version of the UP-A. Regarding feasibility, the attrition rate in this study was 33.33%, a percentage comparable to studies that have evaluated face-to-face transdiagnostic treatments for anxiety and depressive disorders in adults [55], children [56], and adolescents [13]. Other authors have reported lower rates of adherence in studies based on computerized treatments. For example, Stallard et al. [57] reported an adherence rate of 60% for a computerized CBT intervention for anxiety and depression in a sample of children and adolescents. In addition, eight participants in our study completed the post-treatment assessment and had also finished all treatment modules, whereas in other studies with adolescent samples, e.g., Tillfors et al. [58], the average number of completed modules was only 2.9 out of a total of 10 modules. The good adherence shown by participants of the present study may be related to the fact that our treatment included a weekly call from the therapist; the study of Carlbring et al. [59] found that weekly calls increased by 93% the number of participants who finished all modules.

The second aspect of feasibility is the usability, which is the degree to which the participants had a positive experience with the web platform. This was assessed by means of the subscale “experience with the online platform”. Both the adolescents and parents reported a very positive experience with the platform (e.g., mean ratings ranged from 7.25 to 9.38 on a 0 to 10 Likert scale). The third indicator of feasibility is the alliance with the therapist. This was highly rated both by the adolescents (mean ratings ranged from 8.88 to 9.88) and by the parents (mean ratings ranged from 9.38 to 9.88). The levels of therapeutic alliance found in the present study are rather higher than the ones reported by other studies of internet-CBT [60].

Regarding acceptability, the iUP-A and AMTE platform were very positively evaluated by the adolescents and their parents. Users’ satisfaction scores ranged from 8.50 to 9.38 (Likert scale 0 to 10) whereas parents’ satisfaction with the program was also very high, with mean scores ranging from 8 to 9.75 (strong endorsement). These ratings were higher than the ones of similar studies [48]. The participants reported an increase in their coping skills, enjoyment of the program, and reported that they would recommend the program to other adolescents. It is worth noting that a good acceptability is an important issue because expectations about the treatment may affect treatment outcomes.

## 5. Limitations and Future Research

The strengths of this study include performing a comprehensive assessment of feasibility and acceptability, as well as including relevant outcomes in several important domains (general symptoms of anxiety and depression, transdiagnostic constructs, and disorder-specific anxiety and depression symptoms). However, as a preliminary investigation of the iUP-A, it also has several limitations. The first limitation is that the sample size of the current study was too small and may have limited our ability to detect effects of the treatment; however, most of the outcome measures’ effect sizes were large. A second limitation is the lack of a control group since this limits the possibility of establishing causal inferences between the treatment and the changes in the dependent variables. Due to this, it is difficult to determine whether the large effects associated with the treatment may partially reflect a spontaneous improvement over time. Thirdly, the present study did not include follow-up assessments, thereby reducing our ability to draw firm conclusions about the long-term efficacy of the iUP-A. Finally, the current investigation relied on self-report outcome measures.

Although the results are promising, they are very preliminary. These limitations preclude from drawing firm conclusions concerning the efficacy of the iUP-A in the treatment of anxiety and depressive disorders in adolescents. Future research on the efficacy of the iUP-A should address these shortcomings including (a) larger sample size and control conditions in order to be able to establish causal inferences regarding the effect of the intervention vs. the effect due to the passage of time, (b) follow-up assessments to examine if the effect of the treatment is sustained over time, and (c) clinical-rated outcome measures in addition to self-report measures. New research on the efficacy of the iUP-A has important implications for clinical psychology related to the treatment and prevention of anxiety and depression in youth since the UP-A is a consolidated transdiagnostic program for the treatment of emotional disorders. On the other hand, the iUP-A has the advantages of an internet-delivered intervention, thus being a particularly promising intervention program to be applied in the current situation of social isolation linked to COVID-19.

## 6. Conclusions

Overall, the results of the present study suggest that the UP-A delivered via the internet (i.e., the iUP-A) is a promising transdiagnostic CBT program for the treatment of anxiety and depressive disorders in adolescents. Importantly, the iUP-A was not only potentially efficacious but also feasible and acceptable to the participants and their parents. The present study provides preliminary empirical evidence in support of the clinical utility of a Spanish, web-based version of the UP-A (i.e., the iUP-A). This is the first study that provides empirical evidence of the clinical utility of an internet-based version of the UP-A. We found improvements in outcomes associated with several important domains (i.e., anxiety and depression symptoms, as well as vulnerability transdiagnostic constructs), suggesting that the iUP-A is an efficacious treatment reducing general comorbidity of anxiety and depression across emotional disorders (anxiety and depressive disorders). This study also provides empirical evidence concerning the ability of the iUP-A to ameliorate the disorder-specific symptomatology related to anxiety disorders. An innovative contribution of this investigation was to empirically test the effect of the iUP-A on core mechanisms underlying emotional disorders (positive and negative affectivity, anxiety sensitivity, and emotional avoidance). We found that the iUP-A was able to significantly reduce the levels of anxiety sensitivity and emotional avoidance (effect sizes = 1.74 and 1.19, respectively). Finally, the data concerning adherence, usability, therapeutic alliance, and satisfaction with the web platform suggest that the adolescents perceived the treatment as feasible and were highly satisfied with it. We conclude that the iUP-A is feasible and acceptable, as well as potentially efficacious in reducing symptoms (comorbid and disorder-specific) of anxiety and depression as well as underlying vulnerability and maintenance processes.

## Figures and Tables

**Table 1:** Diagnoses or symptoms of the 12 participants.

Principal Diagnosis/Symptoms	Comorbid Diagnoses/Symptoms
Social phobia	Depressive symptoms
Bulimia	Major depressive disorder
Anxiety and depressive symptoms	---
Social phobia	---
Major depressive disorder	Generalized anxiety disorder
Panic disorder with agoraphobia	Obsessive-compulsive disorder
Anxiety symptoms	---
Hypomanic episode–current and past	Separation anxiety disorder; Obsessive-compulsive disorder; Oppositional defiant disorder
Depressive symptoms	---
Panic disorder without agoraphobia	---
Major depressive disorder	Posttraumatic stress disorder
Posttraumatic stress disorder	Agoraphobia; Social phobia; Obsessive-compulsive disorder; Transient TIC Disorder

**Table 2:** Descriptions of the modules (M) included in the AMTE platform.

Module Title	Main Contents
[M1] Building motivation	Obtain three top problems, severity ratings, and a goal for each problem. Discover what motivates the adolescent to change.
[M2] Getting to know your emotions	Psychoeducation about emotions and their function. Understand the three parts of emotional experiences. Learn about emotional behaviors and the cycle of avoidance.
[M3] Enjoy positive activities	Psychoeducation about opposite action and behavioral experiments. Come up with a list of enjoyed activities. Engage the adolescent in behavioral experiments (behavioral activation) for sadness.
[M4] Awareness of your emotional experiences	Introduce the rationale for present-moment awareness and non-judgmental awareness. Practice body scanning. Practice awareness skills when exposed to non-emotional and emotional triggers.
[M5] Learn to be flexible in your thinking	Learn about the concept of “thinking traps” (i.e., cognitive distortions), automatic thoughts, and alternative thoughts. Learn detective thinking and problem-solving skills.
[M6] Cope with your body sensations	Psychoeducation about body sensations, their relationship with intense emotions, and their harmlessness. Conduct exposures to body sensations to learn to tolerate uncomfortable physical feelings.
[M7] Cope with emotional situations	Review the cycle of avoidance and introduce situational emotion exposures. Create an Emotional Behaviors Form to identify relevant exposures. Assign exposures for home learning.
[M8] Maintain your gains	Review skills that have been most useful for each adolescent and make an individualized relapse prevention plan.

**Table 3:** Means (M), standard deviations (SD), Wilcoxon tests, and effect sizes (Cohen’s d) for primary (CDN, EAN, and RCADS-30 total score) and disorder-specific (subscales of the RCADS-30) outcome measures.

Measures	Pre-Treatment *n* = 12		Post-Treatment *n* = 8		Completer Sample Analyses *n* = 8		Intention-to-Treat Analyses ^b^ *n* = 12
	M	SD	M	SD	Z ^a^	d	d
CDN	19.17	11.50	15.25	8.92	1.47	1.14	0.66
EAN	14.50	8.58	10.88	5.82	2.38 **	2.36	1.43
RCADS-30							
Total	32.58	18.43	24.25	9.68	2.38 **	4.14	2.58
MDD	6.42	4.08	4.88	3.23	2.05 *	0.96	0.58
PD	4.83	5.11	1.75	1.98	1.99 *	2.54	1.75
Soc.P	8.58	4.40	8.25	3.49	1.06	0.88	0.52
SAD	1.25	1.54	0.88	1.13	1.84 ^+^	0.74	0.52
GAD	7.33	3.80	5.88	2.10	2.21 *	2.27	1.25
OCD	4.17	4.59	2.63	1.85	1.10	1.32	0.93

Note. ^a^ Z Wilcoxon test based on positive ranks (exact signification, one-tailed). ^b^ Z values are identical to those of the completer sample. CDN = Depression Questionnaire for Children; EAN = Anxiety Scale for Children; GAD = generalized anxiety disorder; MDD = major depressive disorder; OCD = obsessive compulsive disorder; PD = panic disorder; RCADS-30 = Revised Child Anxiety and Depression Scale-30; SAD = separation anxiety disorder; Soc.P = social phobia. ^+^ *p* < 0.10; * *p* < 0.05; ** *p* < 0.01.

**Table 4:** Means (M), standard deviations (SD), Wilcoxon test and effect sizes (Cohen’s d) for transdiagnostic (PANAS, CASI and EASI) and disorder-specific (PDSS-SR, PSWQ-N, and SASC-R) outcome measures.

Measures	Pre-Treatment *n* = 12		Post-Treatment *n* = 8		Completer Sample Analyses *n* = 8		Intention-to-Treat Analyses ^b^ *n* = 12
	M	SD	M	SD	Z ^a^	d	d
PANASN-NA	21.08	2.91	20.75	2.66	0.50	0.53	0.35
PANASN-PA	20.50	3.80	23.38	4.27	1.47 ^+^	−1.13	−0.73
CASI	31.92	8.52	29.00	4.66	2.22 *	2.87	1.74
EASI-A	37.25	16.24	32.13	12.91	1.82 *	2.97	1.19
PDSS-SR	10.58	8.03	2.75	3.49	2.52 **	4.82	2.59
PSWQ-N	32.92	12.92	27.25	5.42	2.39 **	3.40	2.30
SASC-R	37.67	10.82	40.88	5.79	0.42	0.67	0.37

Note. ^a^ Z Wilcoxon test based on positive ranks (exact signification, 1-tailed) (for PANAS-PA, the Z score was based on negative ranks). ^b^ Z values are identical to those of the completer sample. CASI = Childhood Anxiety Sensitivity Index; EASI-A = Emotional Avoidance Strategy Inventory for Adolescents; FNE = Fear of Negative Evaluation; PANASN-NA = Negative Affect scale of the Positive and Negative Affect Schedule for Children and Adolescents; PANASN-PA = Positive Affect scale of the Positive and Negative Affect Schedule for Children and Adolescents; PDSS-SR = Panic Disorder Severity Scale—Self-Report; PSWQ-N = PSWQ-11 questionnaire for children and adolescents; SASC-R = Social Anxiety Scale for Children—Revised. ^+^ *p* < 0.10; * *p* < 0.05; ** *p* < 0.01.

**Table 5:** Descriptive statistics for the items of the FAQ (adolescent version) (*n* = 8).

**Experience with the Online Platform (Range: 0–10)**	**M**	**SD**
How easy has it been for you to use the AMTE online platform?	9.00	1.20
How easy has it been for you to understand what the videos and Dr. AMTE were telling you?	8.50	1.77
How useful has it been for you what Dr. AMTE and the different videos were teaching you?	9.38	0.92
How easy has it been for you to include the AMTE program in your daily routine?	8.50	1.60
To what degree have you been able to do the exercises and home learning assignments without technical or computer problems?	7.25	1.58
To what extent have you applied what you have learned with AMTE to your real life?	8.50	0.93
**Satisfaction with the Program (Range: 0–10)**	**M**	**SD**
How much did you learn in this program?	8.75	1.58
How effective was this program in helping you cope with your problems?	8.86	1.07
How much did you enjoy doing this program?	8.50	1.20
To what extent would you recommend the program to other adolescents?	9.38	0.74
What was your ability to cope with emotions before the program?	4.13	1.64
What was your ability to cope with emotions after the program?	8.25	1.39
Ability to cope with emotions after the program minus ability before	4.13	1.96
**Therapeutic Alliance (Range: 0–10)**	**M**	**SD**
How much has your therapist helped you deal with your top problems?	8.88	1.46
How appreciated by your therapist have you felt?	9.50	0.76
To what extent have you felt that you and your therapist respected each other?	9.88	0.35
To what extent have you agreed with your therapist on what things were important for you to work to overcome?	9.50	1.07
To what extent have you felt that your therapist cared about you?	9.38	0.92
How correct do you think the way you and your therapist have worked to solve your problems has been?	9.38	0.92

**Table 6:** Descriptive statistics for the items of the FAQ (parent version) (*n* = 8).

**Experience of the Adolescent with the Online Platform (Range: 0–10)**	**M**	**SD**
How easy has it been for your son/daughter to use the AMTE online platform?	8.50	1.31
How easy has it been for your child to understand what the videos and Dr. AMTE were telling them?	9.25	1.04
How useful has it been for your child what Dr. AMTE and the different videos were teaching them?	8.88	1.36
How easy has it been for your child to include the AMTE program in their daily routine?	8.63	1.41
To what degree has your child been able to do the exercises and home learning assignments without technical or computer problems?	8.63	1.06
To what extent has your child applied what he/she has learned with AMTE to their real life?	8.38	1.60
**Experience with the Parent’s Section of the Online Platform**	***n* (%) [yes]**	
Have you ever logged in to the parent’s section of the AMTE platform?	7 (87.50%)	
To what extent has the parent’s section of the platform helped you to help your son/daughter during treatment? (range: 0–10)	7.29	2.81
**Satisfaction with the Program (Range: 0–10)**	**M**	**SD**
How much has your child learned in this program?	9.14	0.69
How effective was this program in helping your child cope with their problems?	9.14	1.07
How much has your child enjoyed doing this program?	8.00	2.27
To what extent would you recommend the program to other adolescents?	9.75	0.71
What was the ability of your child to cope with emotions before the program?	4.63	2.00
What was the ability of your child to cope with their emotions after the program?	8.00	1.51
Ability to cope with emotions after the program minus ability before	3.38	1.51
**Therapeutic Alliance (Range: 0–10)**	**M**	**SD**
How much has the therapist helped your child deal with their top problems?	9.38	0.92
How appreciated by the therapist has your child felt?	9.88	0.35
To what extent have you felt that you and the therapist respected each other?	9.88	0.35
To what extent have you agreed with the therapist on what things were important for your son/daughter to work to overcome?	9.75	0.46
To what extent have you felt that the therapist cared about your child?	9.88	0.35
How correct do you think the AMTE’s approach to solving your child problems has been?	9.75	0.46
